# A cross-sectional retrospective analysis of the regionalization of complex surgery

**DOI:** 10.1186/1471-2482-14-55

**Published:** 2014-08-16

**Authors:** James Studnicki, Christopher Craver, Christopher M Blanchette, John W Fisher, Sara Shahbazi

**Affiliations:** 1 Department of Public Health Sciences, College of Health and Human Services, University of North Carolina, Charlotte, NC, USA; 2 College of Health and Human Services, University of North Carolina, Charlotte, NC, USA

**Keywords:** Surgical complexity, Surgical outcomes, Regionalization of surgical services

## Abstract

**Background:**

The Veterans Health Administration (VHA) system has assigned a surgical complexity level to each of its medical centers by specifying requirements to perform standard, intermediate or complex surgical procedures. No study to similarly describe the patterns of relative surgical complexity among a population of United States (U.S) civilian hospitals has been completed.

**Methods:**

Design: single year, retrospective, cross-sectional.

Setting/Participants: the study used Florida Inpatient Discharge Data from short-term acute hospitals for calendar year 2009. Two hundred hospitals with 2,542,920 discharges were organized into four quartiles (Q 1, 2, 3, 4) based on the number of complex procedures per hospital. The VHA surgical complexity matrix was applied to assign relative complexity to each procedure. The Clinical Classification Software (CCS) system assigned complex procedures to clinically meaningful groups. For outcome comparisons, propensity score matching methods adjusted for the surgical procedure, age, gender, race, comorbidities, mechanical ventilator use and type of admission.

Main Outcome Measures: in-hospital mortality and length-of-stay (LOS).

**Results:**

Only 5.2% of all inpatient discharges involve a complex procedure. The highest volume complex procedure hospitals (Q4) have 49.8% of all discharges but 70.1% of all complex procedures. In the 133,436 discharges with a primary complex procedure, 374 separate specific procedures are identified, only about one third of which are performed in the lowest volume complex procedure (Q1) hospitals. Complex operations of the digestive, respiratory, integumentary and musculoskeletal systems are the least concentrated and proportionately more likely to occur in the lower volume hospitals. Operations of the cardiovascular system and certain technology dependent miscellaneous diagnostic and therapeutic procedures are the most concentrated in high volume hospitals. Organ transplants are only done in Q4 hospitals. There were no significant differences in in-hospital mortality rates and the longest lengths of stay were found in higher volume hospitals.

**Conclusions:**

Complex surgery in Florida is effectively regionalized so that small volume hospitals operating within the range of complex procedures appropriate to their capabilities provide no increased risk of post surgical mortality.

## Background

Any evaluation of surgical outcomes must consider the influence of the relative complexity of the surgical procedures. Surgical complexity has been conceptualized and measured largely by addressing both intraoperative factors and patient risk factors. Intraoperative factors considered to influence surgical complexity include blood loss, duration of the surgery, technical expertise required of the surgeon, invasiveness of the procedure, organ system involvement, existing pathology and required technical equipment [1-14]. Patient characteristics, most notably age and co-morbid conditions, are strongly associated with operative complexity [5,11,12,15-20]. Existing research consistently relates procedural complexity to multiple outcomes such as increased mortality and morbidity, greater length of stay, increased likelihood of a readmission, longer time in intensive care and higher overall costs [1,3,4,8,9,12,19,21-30].

While hospital and surgeon volumes have been revealed to be important determinants of surgical outcomes, the volume-outcome relationship deserves particular emphasis for complex procedures since they are performed in small numbers overall and typically constitute only a small proportion of hospital total caseloads. Policy experts, insurance companies, coalitions of purchasing groups and others advocate that patients needing complex surgical procedures should be referred to hospitals with the best outcomes, particularly high volume facilities [1,9,26,29,31].

Perhaps the best example of a large-scale application of these concepts comes from the VHA and its National Surgical Quality Improvement Program (NSQIP), which developed a validated risk-adjusted model to predict surgical outcomes. The predictive model included a measure of surgical complexity [32]. This effort led the VHA to undertake a major restructuring of its medical centers based upon the level of complexity of the surgical procedures provided. In 2010, the VHA issued directive 2010–018, Facility Infrastructure Requirements to Perform Standard, Intermediate, or Complex Surgical Procedures [33]. The NSQIP Operative Complexity Workgroup created a Surgical Complexity Matrix to classify Current Procedural Terminology (CPT) codes of surgical procedures into one of three categories: standard, intermediate, or complex [15]. Based on these analyses, each veterans hospital was assigned a surgical complexity level based on its clinical capabilities, facilities, equipment, caseload and staffing considerations. Individual VHA facilities faced with providing a non-emergent procedure beyond their designated level of complexity were charged with ensuring the safe and timely transfer of the patient to an appropriate VHA facility. It is important to emphasize that policy decisions and management procedures are highly centralized in the VHA system. While data from the Nationwide Inpatient Sample (NIS) indicates that 71 percent of patients in the U.S. reside in healthcare referral regions with high volume hospitals for Coronary Artery Bypass Graft (CABG), suggesting a high degree of regionalization for that specific procedure, no existing study has attempted to characterize the degree of regionalization for all complex surgeries in a large study population of civilian hospitals which are not centrally controlled as in the VHA. Therefore this research sought to describe the distribution of all hospital admissions characterized by their surgical complexity across a population of autonomous, non-governmental hospitals and to assess the implications of this distribution on complex surgical outcomes.

## Methods

### Study design

A retrospective, single year cross-sectional study was used to examine the distribution and selected outcomes associated with the relative complexity of surgical discharges in a study population of 200 hospitals. The hospitals were organized into quartiles based upon the number of complex surgical procedures performed in each hospital in order to provide a base analytic framework.

### Study population/setting

The study used Florida Inpatient Discharge Data for the calendar year 2009 from the Florida Agency for Health Care Administration. Florida is a state with a large, racially and ethnically diverse population; a large number of non-governmental short-term acute hospitals; and a number of major metropolitan areas as well as many rural counties. This study (protocol 10-05-28) has been approved (exempt category 4) by the Institutional Review Board (IRB) for Research with Human Subjects, University of North Carolina at Charlotte. Independent variables of interest included patient age, gender, race, ethnicity, length of stay, number of procedures per patient, days from admission to procedure, type of admission, and hospital bed size and ownership. Principal and secondary diagnosis and procedure codes were used to derive two co-morbidity measures and to assign levels of complexity to each discharge with a surgical procedure.

### Surgical complexity

We applied the Surgery Complexity Matrix developed by the VHA NSQIP Operative Complexity Workgroup to identify all discharges with a primary surgical procedure as standard, intermediate or complex [32]. Commercial software from Context Healthcare, Inc. was used to cross reference the International Classification of Diseases, Ninth Revision, Clinical Modification (ICD-9-CM) codes from the inpatient data source with the CPT-4 codes used in the VHA complexity matrix, allowing the examination of proportional surgical complexity at the hospital level.

### Co-morbidity measures

To adjust for patient case mix, we used version 3.5 (January, 2010) of the co-morbidity software developed by Elixhauser and colleagues and distributed by Agency for Healthcare Research & Quality (AHRQ) [17]. The output of this system is a count of the 30 binary variables (co-morbid conditions) found in each discharge record. Further, we used a modification of the Elixhauser co-morbidity measures to assign a single numeric score to each discharge derived by weighting the relative importance of each of the 30 co-morbidities [34].

### Main outcome measures

ICD-9-CM Codes and the CCS System were used to identify all of the complex surgical procedures performed, and to allocate the complex surgical procedures across the hospital quartiles into 16 clinically meaningful categories. Paired hospital quartile differences were assessed for in-hospital mortality and length-of-stay for matched surgical procedures.

### Statistical analyses

Quartile differences in patient and hospital characteristics, as well as discharge complexity volumes, were assessed using Chi Square (categorical variables) and F-test and ANOVA (continuous variables).

To test the association of surgical complexity volume and mortality and LOS, we performed a propensity score match to account for patient differences in the surgical procedure, co-morbidity profile and mechanical ventilator use, as well as demographic characteristics including age, sex, race, and type of admission. Due to the extreme likelihood of death, patients diagnosed with acute renal failure were removed from the study population.

To accomplish the patient match a greedy algorithm was used to match cases to controls. The greedy match algorithm is frequently used for its ability to reduce the number of incomplete and inexact matches [35,36]. This algorithm matches cases with the highest precision match first and continues to perform matches until no additional matches are found. Sensitivity analyses were conducted to insure the integrity of the match.

Patient cohort matches were performed hierarchically between each quartile using the lower volume quartile as a base. Thus quartile1 patients were matched discreetly to similar patients in quartiles 2, 3 and 4. Quartile 2 patients were matched to quartiles 3 and 4, and quartile 3 patients were only matched to quartile 4 patients. This process yielded six unique patient cohorts for comparison.

Using these matched cohorts, bivariate statistical methods were used to test the difference between the two patient populations. Chi-Square was used to test the mortality outcome, while Wilcoxon ranked sums was used for the length-of-stay outcome. All statistical analyses were performed using [*SAS/STAT*] software, Version [9.2 of the SAS System for [Windows]. Copyright © 2002–2008 SAS Institute Inc. SAS and all other SAS Institute Inc. product or service names are registered trademarks or trademarks of SAS Institute, Inc., Cary, NC, USA.

## Results

### Total discharges by procedure complexity

There were just over 2.5 million discharges from 200 short-term acute hospitals in Florida in 2009. Across all hospitals, 1,044,975 (41.1%) of these discharges involved no procedure and another 453,261 (17.8%) involved a non-surgical procedure. Of the discharges which involved a surgical procedure, 336,280 (14.4%) were considered to be of standard complexity, 504, 495 (19.8%) of intermediate complexity, and 133, 436 (5.2%) were in the complex category.

As complex procedure volumes increase across the four hospital quartiles, the percent of discharges with complex, intermediate, and non-surgical procedures increase; the percent of discharges with no procedure decreases; and, the percent of discharges with procedures of standard complexity is relatively uniform.

Quartile 1 hospitals discharged only 6.8% of all patients, an average of 3,445 discharges per hospital. Of these Q1 discharges, 56.1% had no procedure and only 1.5% involved a complex procedure. Q1 hospitals averaged only 51 complex procedures annually. By contrast quartile 4 hospitals discharged 49.8% of all patients, an average of 25,333 discharges per hospital. Of these Q4 discharges, 35.6% had no procedure and 7.4% involved a complex procedure. Q4 hospitals averaged 1,871 complex procedures annually. Therefore, Q4 hospitals had, on average, more than 7 times the number of discharges annually than Q1 hospitals but more than 36 times the number of discharges with complex procedures (Table 1).

**Table 1:** **Florida (2009) discharges by procedure complexity**^**a**^

**Discharges with procedures**								
**QTR s**	**Non-surgical**	**STAND**	**INT.M**	**CO**	**Un-assigned**	**Total procedures**	**No procedure**	**Total procedures**
QTR 1	19368	24479	25117	2568	4172	75704	96561	172265
R%	(11.2)	(14.2)	(14.6)	(1.5)	(2.4)	(43.9)	(56.1)	(100.0)
C%	(4.3)	(7.3)	(5.0)	(1.9)	(5.9)	(5.1)	(9.2)	(6.8)
QTR 2	70291	56988	81055	9969	11188	229491	201214	430705
	(16.3)	(13.2)	(18.8)	(2.3)	(2.6)	(53.3)	(46.7)	(100.0)
	(15.5)	(16.9)	(16.1)	(7.5)	(15.9)	(15.3)	(19.3)	(16.9)
QTR 3	109114	92730	131929	27364	16380	377517	295781	673298
	(16.2)	(13.8)	(19.6)	(4.1)	(2.4)	(56.1)	(43.9)	(100.0)
	(24.1)	(27.6)	(26.1)	(20.5)	(23.2)	(25.2)	(28.3)	(25.5)
QTR 4	254488	162083	266394	93535	38733	815233	451419	1266652
	(20.1)	(12.8)	(21.0)	(7.4)	(3.1)	(64.4)	(35.6)	(100.0)
	(56.1)	(48.2)	(52.8)	(70.1)	(55.0)	(54.4)	(43.2)	(49.8)
Total	453261	336280	504495	133436	70473	1497945	1044975	2542920
	(17.8)	(14.4)	(19.8)	(5.2)	(2.8)	(58.9)	(41.1)	
	(100.0)	(100.0)	(100.0)	(100.0)	(100.0)	(100.0)	(100.0)	(100.0)

*Abbreviations:R%* Row Percentage, *C%* Column Percentage, *QTR* Quartile, *Stand* Standard, *Int. M* Intermediate, *Co* Complex.

^a^Expressed as number (percentage) unless otherwise indicated. Percentages may not total 100 because of rounding.

### The distribution of complex discharges

Of the 133,436 complex procedure discharges, Q1 accounted for only 1.9%, Q2 7.5%, Q3 20.5%, and Q4 70.1%. A total 374 specific complex surgical procedures were identified in the transformation from CPT to ICD-9-CM codes provided in the discharge data. Of these, 126 were performed in the lowest volume hospitals of Q1, 88 more were added in Q2 (total 214), 71 in Q3 (total 285) and another 89 procedures performed only in Q4 (total 374). Clearly, as hospitals provide a higher volume of complex procedures, the range of procedures also widens.

**Figure 1:**
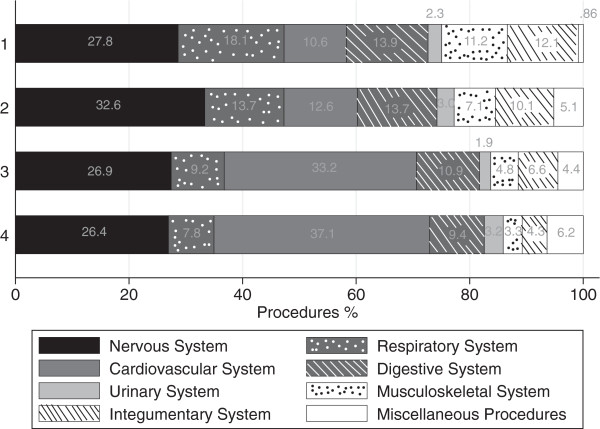
Distribution of complex surgical procedures by quartiles.

**Figure 2:**
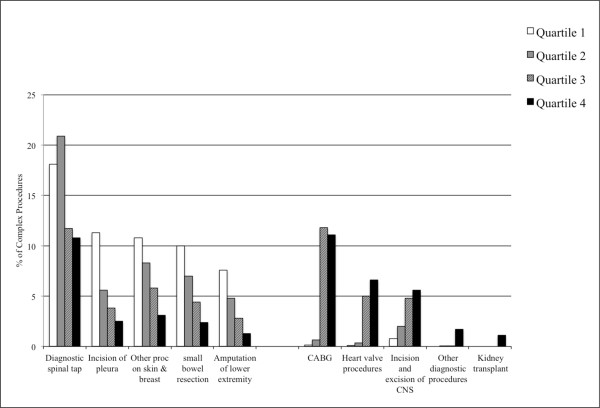
Percent of total complex procedures inside quartiles represented by specific procedures.

The CCS allocated the complex surgical procedures across the quartiles into 16 clinically meaningful categories. Eight of the CCS procedure categories did not have enough cases to represent at least 1% of the complex procedures within any quartile. The remaining 8 categories accounted for 97% of the complex procedures.In four categories, the CCS procedure groups represented a smaller percentage of total complex procedures within the quartile as the overall volume increased: operations of the digestive system; operations of the respiratory system; operations of the integumentary system; and, operations of the musculoskeletal system. Viewed in another way, complex procedures in these categories are relatively decentralized and are proportionately more likely to occur in smaller rather than larger hospitals. Operations of the cardiovascular system, by contrast, represent higher percent of total complex procedures within the quartiles as volume increased (Figure 1).Similarly, at the specific CCS procedure level, it is possible to see this same pattern. Small bowel resection and amputation of a lower extremity, for example, represent a higher percent of the complex surgeries in lower volume hospitals. Heart valve procedures and CABG, as well as incision and excision of the Central Nervous System (CNS), represent a higher percent of complex surgeries in the higher volume hospitals. Organ transplant and certain other diagnostic and therapeutic procedures are limited to quartile 4 hospitals (Figure 2).

### Mortality and length of stay outcomes

The six matched patient cohorts showed no statistically significant differences in in-hospital mortality (Table 2). It is worth emphasizing that the matched quartile comparisons were adjusted for the types of procedure, comorbidities and patient age, race, sex and type of admission. Therefore, the mortality comparisons between the smallest and largest hospitals (quartiles 1 and 4) only involved the narrower range of complex procedures being performed in the smallest hospitals. For these procedures, the higher total volumes of all complex procedures occurring in the largest hospitals provide no mortality advantage.

**Table 2:** Propensity score match comparison: hospital mortality by complex surgery quartile

**Mortality**	**Matched**^**a**^	**Based**^**b**^	**Comparison**	**Base (%)**	**Comparison (%)**	**Significance**	
**Quartile comparison**	**Population size**	**Mortalities**	**Mortalities**	**Mortality R**	**Mortality R**	**Chi**^**2**^	**Pr > |Chi**^**2**^
1 vs. 4	2916	20	15	1.4	1.0	0.72	0.39
1 vs. 3	2476	12	15	1.0	1.2	0.33	0.56
1 vs. 2	2152	11	8	1.0	0.7	0.47	0.48
2 vs. 4	11692	49	68	0.8	1.2	3.11	0.07
2 vs. 3	9798	44	40	0.9	0.8	0.19	0.66
3 vs. 4	16205	97	222	2.4	2.7	1.51	0.21

*Abbreviation:Mortality R* Mortality Rate.

^a^Matched Population base divided by two = quartile population.

^b^Base mean represents the average mortality rate for the base quartile.

Length-of-stay for the matched patient cohorts shows an increasing length of stay as the volume of complex procedures increases, with three exceptions. The LOS differences between quartiles 1 and 2 and 1 and 3 are not significant. In contrast, the LOS difference between quartiles 2 and 3 is significant but reversed (i.e., LOS in quartile 2 is larger than quartile 3). The LOS gap between the smallest and largest hospitals is about a full day (Table 3).

**Table 3:** Propensity score match comparison: median length of stay by complex surgery quartile

**Length of stay**	**Matched**^**a**^	**Based**^**b**^			**Comparison**			**Mean diff**	**Significance**^**d**^	
**Quartile comparison**	**Population size**	**Mean**	**Std**	**Median**	**Mean**	**Std**	**Median**	**Mean diff **^**c**^	**Z Value**	**Pr > |z|**
1 vs. 4	2916	5.5	5.53	4	6.4	7.43	4	0.98	2.94	0.00
1 vs. 3	2476	5.7	6.53	4	6.2	5.20	4	0.56	1.73	0.08
1 vs. 2	2152	5.2	5.76	4	5.3	5.53	4	0.07	-0.08	0.93
2 vs. 4	11692	5.5	8.76	3	6.2	10.54	4	0.75	-5.43	<.001
2 vs. 3	9798	5.3	8.09	3	5.0	6.67	3	-0.29	-2.05	0.04
3 vs. 4	16205	5.8	7.47	4	6.2	8.85	4	0.41	4.98	<.001

^a^Matched Population base divided by two = quartile population.

^b^Base median represents the median LOS for the base quartile.

^c^Median difference = comparison median-base median.

^d^Significance test is Wilcoxon Rank Sum (2-sided).

## Discussion

Complex surgeries in Florida hospitals represent only about five percent of total discharges but they are highly concentrated, disproportionate even to the relative concentration of total discharges. There is also a hierarchy of regionalization in which certain complex procedures are performed only in hospitals that attain complex surgery volume thresholds. As a result, the overall composition of complex surgery caseloads vary among hospitals as volume increases. From a quality perspective, these results provide evidence that both formal and informal methods of regionalization in Florida are generally allocating complex surgeries among hospitals in a manner consistent with the capabilities of each facility.

Private sector efforts to regionalize or concentrate complex surgeries have been less comprehensive and much more “procedure specific” than the VHA program. Further, approaches to enhancing volumes for complex surgeries can be characterized as either formal or informal. Formal regionalization can be inferred to mean a deliberate geographic centralization of surgical services via governmental regulatory authority, such as state Certificate-of-Need (CON) laws [37]. Informal regionalization has been defined as the concentration of select patient populations at specific local centers as a result of selective, historic, or de facto referral patterns to those centers by providers [38]. The selective referral of patients resulting from informal regionalization is based on decentralized decisions by individual providers and natural market dynamics and is not mandated by formal legal or administrative organizations.

The characteristics of complex surgeries and the methods utilized in this analysis suggest caution in the interpretation of these results. With only a few exceptions (e.g. CABG), complex surgical procedures occur infrequently and therefore represent a low volume overall. This means that, for certain procedures, even the highest volume hospitals will perform the procedure a modest number of times. For analyses of the relationship between specifically defined procedure volumes and outcomes, the quartile comparison approach utilized in this analysis would be inadequate since most of the hospitals in these comparisons would likely be drawn from the highest volume hospitals in quartiles 3 and 4. Another important limitation in this study involves the use of the propensity score match algorithm which is applied to all complex procedures in the base (i.e. lower volume) quartile. Because of the very low volume of many complex procedures performed in quartiles 1 and 2, and the multiple covariates utilized in the matching, many complex procedures were not included in the volume/outcome comparisons. A major focus of quality monitoring of complex procedures should be on the procedures which are rarely done in the smallest volume hospitals, but nonetheless do infrequently occur. Our analytical approach was unable to address this problem.

## Conclusions

Complex surgery in Florida is effectively regionalized likely as the results of both formal and informal influences. Small volume hospitals operating within the range of complex procedures appropriate to their capabilities provide no increased risk of post surgical mortality.

## Abbreviations

AHRQ: Agency for healthcare research & quality; CABG: Coronary artery bypass grafting; CCS: Clinical classification software; CNS: Central nervous system; CON: Certificate-of-need; CPT: Current procedural terminology; ICD-9-CM: International classification of diseases, ninth revision, clinical modification; NIS: Nationwide inpatient sample; NSQIP: National surgical quality improvement program; LOS: Length of stay; US: United States; VHA: Veterans health administration.

## Competing interests

The authors declare that they have no competing interests.

## Authors’ contributions

JS: Integrity of the work as a whole. JS and CC were involved in the conception and design of the study. JS and JWF were responsible for the acquisition of data. JS, CMB, CC, and SS performed the analysis and interpretation of data. JS and CC wrote the draft of the manuscript. All authors critically reviewed and gave final approval to the manuscript.

## Pre-publication history

The pre-publication history for this paper can be accessed here:

http://www.biomedcentral.com/1471-2482/14/55/prepub
